# *N*,*N*-Bis(diphenyl­phosphan­yl)cyclo­­penta­namine

**DOI:** 10.1107/S1600536810048907

**Published:** 2010-11-27

**Authors:** Ilana Engelbrecht, Hendrik G. Visser, Andreas Roodt

**Affiliations:** a Department of Chemistry, University of the Free State, PO Box 339, Bloemfontein 9300, South Africa

## Abstract

The coordination around the N atom in the title compound, C_29_H_29_NP_2_, shows an almost planar geometry, defined by the attached P and C atoms, in order to accomodate the steric bulk of the phenyl rings. The distortion of the trigonal–pyramidal geometry of the N atom is illustrated by the bond angles ranging between 115.22 (12) and 121.76 (9)°. The P atoms present a pyramidal environment with bond angles ranging from 100.62 (9) to 104.71 (8)°. One of the C atoms in the cyclo­pentyl ring displays a 0.822 (4):0.178 (4) positional disorder. Within the crystal structure, intra­molecular C—H⋯P hydrogen bonds together with inter- and intra­molecular C—H⋯π inter­actions link the mol­ecules into a supra­molecular two-dimensional network.

## Related literature

For similar structures, see: Keat *et al.* (1981 ▶); Cotton *et al.* (1996 ▶); Fei *et al.* (2003 ▶); Cloete *et al.* (2008 ▶, 2009 ▶, 2010 ▶); Engelbrecht *et al.* (2010 ▶).
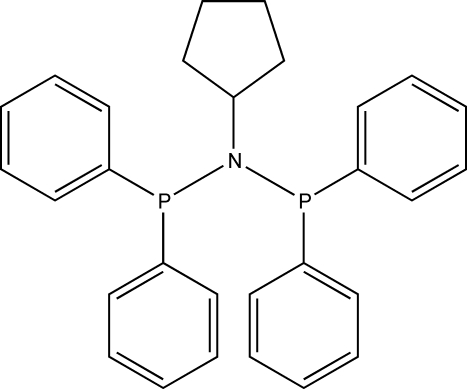


## Experimental

### 

#### Crystal data

C_29_H_29_NP_2_
*M* *_r_* = 453.47
Triclinic, 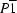

*a* = 8.803 (5) Å
*b* = 11.166 (4) Å
*c* = 12.685 (5) Å
α = 97.144 (4)°
β = 101.261 (5)°
γ = 99.707 (5)°
*V* = 1189.3 (10) Å^3^
*Z* = 2
Mo *K*α radiation
μ = 0.2 mm^−1^
*T* = 100 K
0.19 × 0.13 × 0.08 mm

#### Data collection

Bruker X8 APEXII 4K Kappa CCD diffractometer
Absorption correction: multi-scan (*SADABS*; Bruker, 2004 ▶) *T*_min_ = 0.963, *T*_max_ = 0.983
12794 measured reflections
5817 independent reflections
4333 reflections with *I* > 2σ(*I*)
*R*_int_ = 0.035

#### Refinement

*R*[*F* ^2^ > 2σ(*F* ^2^)] = 0.045
*wR*(*F* ^2^) = 0.106
*S* = 1.06
5817 reflections
293 parameters
H-atom parameters constrained
Δρ_max_ = 0.34 e Å^−3^
Δρ_min_ = −0.33 e Å^−3^

### 

Data collection: *APEX2* (Bruker, 2010 ▶); cell refinement: *SAINT-Plus* (Bruker, 2004 ▶); data reduction: *SAINT-Plus*; program(s) used to solve structure: *SHELXS97* (Sheldrick, 2008 ▶); program(s) used to refine structure: *SHELXL97* (Sheldrick, 2008 ▶); molecular graphics: *DIAMOND* (Brandenburg & Putz, 2005 ▶); software used to prepare material for publication: *WinGX* (Farrugia, 1999 ▶).

## Supplementary Material

Crystal structure: contains datablocks global, I. DOI: 10.1107/S1600536810048907/bg2375sup1.cif

Structure factors: contains datablocks I. DOI: 10.1107/S1600536810048907/bg2375Isup2.hkl

Additional supplementary materials:  crystallographic information; 3D view; checkCIF report

## Figures and Tables

**Table 1:** Hydrogen-bond geometry (Å, °) *Cg*1 is the centroid of the C11–C16 ring.

*D*—H⋯*A*	*D*—H	H⋯*A*	*D*⋯*A*	*D*—H⋯*A*
C2—H2*B*⋯P2	0.99	2.82	3.269 (2)	108
C5*A*—H5*A*1⋯P2	0.99	2.82	3.202 (3)	104
C12—H12⋯*Cg*1	0.95	2.72	3.667 (2)	174
C3*A*—H3*A*2⋯*Cg*1^i^	0.99	2.89	3.811 (2)	155

Symmetry code: (i) 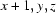.

**Fig. 1.**
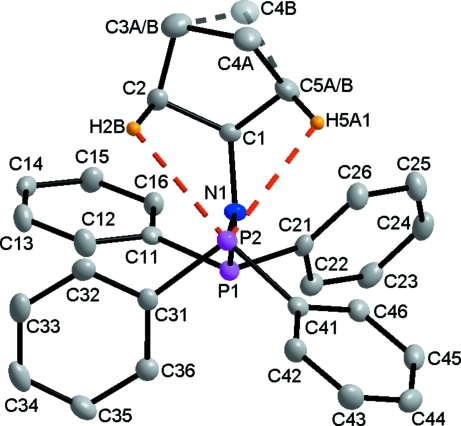
Molecular structure of the title compound. Displacement ellipsoids are drawn at the 50% probability level. Dashed lines denote the minor disordered atoms. Only applicable hydrogen atoms with relevance to C—H···P intramolecular hydrogen bonds are indicated.

**Fig. 2.**
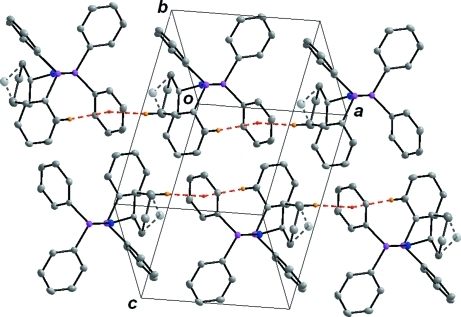
The crystal packing of the title compound showing the two-dimensional network. C—H···π interactions are shown as dashed lines.

## supplementary crystallographic information

### Comment

Diphosphinoamine (PNP) ligands form part of ongoing research in ethylene tetramerization catalyst systems. In the title compound, C_29_H_29_NP_2_, all bond distances and angles fall within the range for similar complexes (Keat *et al.*, 1981; Cotton *et al.*, 1996; Fei *et al.*, 2003; Cloete *et al.*, 2008, 2009, 2010; Engelbrecht *et al.*, 2010) The N(P_2_C) group is almost planar, with the central N displaced by -0.120 (2)Å from the plane defined by the remaining three atoms (P1, P2, C1). The distorted trigonal-pyramidal geometry around the N atom is further illustrated by the bond angles ranging between 115.22 (12)° and 121.76 (9)°. The diphenylphosphino groups are staggered relative to the PNP backbone and form with each other dihedral angles of 68.84 (4)° (C11 and C21, bonded to P1) and 68.43 (4)° (C31 and C41, bonded to P2). The geometry around the phosphorous atoms is that of a distorted triangular pyramid, with C—P—C angles in the range 100.62 (9)° - 101.65 (8)° and N—P—C angles with a 101.65 (8)° - 104.71 (8)° span. One carbon atom in the cyclopentyl ring is disordered over two positions in a 0.822 (4):0.178 (4) ratio (Fig 1). There are some C—H···P intramolecular H-bonds as well as a few C—H···π interactions which contribute to the supramolecular aggregation (Table 1, Figure 2).

### Experimental

Cyclopentylamine (0.010 mol, 1.00 ml) was dissolved in dichloromethane (30 ml) after which the solution was placed on an ice bath. Triethylamine (0.030 mol, 4.21 ml) was added to the solution while stirring. Chlorodiphenylphosphine (0.020 mol, 3.70 ml) was slowly added to the reaction mixture. The ice bath was removed after 1 h and the reaction mixture was allowed to stir at room temperature for a further 12 h. The dichloromethane was removed under reduced pressure. A mixture of hexane (20 ml) and toluene (2 ml) was added to the remaining white powder and was passed through a column containing neutral activated alumina (35 g). The solvent of the eluent was removed under reduced pressure and the white precipitate was collected. Single colourless crystals suitable for X-ray crystallography were obtained from recrystallization from methanol. (yield: 1.010 g, 22%)

### Refinement

The methine, methylene and aromatic H atoms were placed in geometrically idealized positions at C—H = 1.00, 0.99 and 0.95 Å, respectively and constrained to ride on their parent atoms, with *U*_iso_(H) = 1.2*U*_eq_(C). The highest peak is located 0.48Å from N1 and the deepest hole is situated 0.39Å from H3B1.

### Crystal data

**Table:**

C_29_H_29_NP_2_	*Z* = 2
*M**_r_* = 453.47	*F*(000) = 480
Triclinic, *P*1	*D*_x_ = 1.266 Mg m^−^^3^
Hall symbol: -P 1	Mo *K*α radiation, λ = 0.71073 Å
*a* = 8.803 (5) Å	Cell parameters from 2872 reflections
*b* = 11.166 (4) Å	θ = 2.7–28.0°
*c* = 12.685 (5) Å	µ = 0.2 mm^−^^1^
α = 97.144 (4)°	*T* = 100 K
β = 101.261 (5)°	Cuboid, white
γ = 99.707 (5)°	0.19 × 0.13 × 0.08 mm
*V* = 1189.3 (10) Å^3^	

### Data collection

**Table:**

Bruker X8 APEXII 4K Kappa CCD diffractometer	5817 independent reflections
Radiation source: fine-focus sealed tube	4333 reflections with *I* > 2σ(*I*)
graphite	*R*_int_ = 0.035
ω and φ scans	θ_max_ = 28.3°, θ_min_ = 3.1°
Absorption correction: multi-scan (*SADABS*; Bruker, 2004)	*h* = −11→11
*T*_min_ = 0.963, *T*_max_ = 0.983	*k* = −14→14
12794 measured reflections	*l* = −16→16

### Refinement

**Table:**

Refinement on *F*^2^	Primary atom site location: structure-invariant direct methods
Least-squares matrix: full	Secondary atom site location: difference Fourier map
*R*[*F*^2^ > 2σ(*F*^2^)] = 0.045	Hydrogen site location: inferred from neighbouring sites
*wR*(*F*^2^) = 0.106	H-atom parameters constrained
*S* = 1.06	*w* = 1/[σ^2^(*F*_o_^2^) + (0.0343*P*)^2^ + 0.5079*P*] where *P* = (*F*_o_^2^ + 2*F*_c_^2^)/3
5817 reflections	(Δ/σ)_max_ = 0.015
293 parameters	Δρ_max_ = 0.34 e Å^−^^3^
0 restraints	Δρ_min_ = −0.33 e Å^−^^3^

### Special details

**Table:**

Experimental. The intensity data were collected on a Bruker X8 ApexII 4 K Kappa CCD diffractometer using an exposure time of 60 s/frame. A total of 1148 frames were collected with a frame width of 0.5° covering up to θ = 28.27° with 98.7% completeness accomplished. Spectroscopy data: ^1^H NMR (300 MHz, CD_2_Cl_2_): δ = 1.3 to 1.9 (m, 8H, 4 *x* CH_2_), 3.8 (m, 1H, CH), 7.3 to 7.4 (m, 20H, Ar); ^31^P NMR (121 MHz, CD_2_Cl_2_): δ = 50.6 (*s*).
Geometry. All s.u.'s (except the s.u. in the dihedral angle between two l.s. planes) are estimated using the full covariance matrix. The cell s.u.'s are taken into account individually in the estimation of s.u.'s in distances, angles and torsion angles; correlations between s.u.'s in cell parameters are only used when they are defined by crystal symmetry. An approximate (isotropic) treatment of cell s.u.'s is used for estimating s.u.'s involving l.s. planes.
Refinement. Refinement of *F*^2^ against ALL reflections. The weighted *R*-factor *wR* and goodness of fit *S* are based on *F*^2^, conventional *R*-factors *R* are based on *F*, with *F* set to zero for negative *F*^2^. The threshold expression of *F*^2^ > 2σ(*F*^2^) is used only for calculating *R*-factors(gt) *etc*. and is not relevant to the choice of reflections for refinement. *R*-factors based on *F*^2^ are statistically about twice as large as those based on *F*, and *R*- factors based on ALL data will be even larger.

### Fractional atomic coordinates and isotropic or equivalent isotropic displacement parameters (Å^2^)

**Table:**

	*x*	*y*	*z*	*U*_iso_*/*U*_eq_	Occ. (<1)
C1	0.91678 (19)	0.34252 (17)	0.75151 (15)	0.0160 (4)	
H1	0.9698	0.278	0.7211	0.019*	
C2	0.9126 (2)	0.44037 (18)	0.67528 (16)	0.0198 (4)	
H2A	0.9168	0.4043	0.6008	0.024*	
H2B	0.8154	0.4746	0.6716	0.024*	
C3A	1.0595 (2)	0.5401 (2)	0.72654 (18)	0.0283 (5)	0.822 (4)
H3A1	1.0467	0.6203	0.7042	0.034*	0.822 (4)
H3A2	1.1549	0.5173	0.706	0.034*	0.822 (4)
C4A	1.0695 (3)	0.5448 (2)	0.8501 (2)	0.0252 (6)	0.822 (4)
H4A1	1.1775	0.5823	0.8926	0.03*	0.822 (4)
H4A2	0.9939	0.5922	0.8748	0.03*	0.822 (4)
C5A	1.0262 (2)	0.41072 (18)	0.86124 (16)	0.0222 (4)	0.822 (4)
H5A1	0.9707	0.4024	0.9215	0.027*	0.822 (4)
H5A2	1.1226	0.3755	0.8771	0.027*	0.822 (4)
C3B	1.0595 (2)	0.5401 (2)	0.72654 (18)	0.0283 (5)	0.178 (4)
H3B1	1.1228	0.5556	0.6714	0.034*	0.178 (4)
H3B2	1.0268	0.6177	0.7509	0.034*	0.178 (4)
C4B	1.1460 (13)	0.5064 (11)	0.8099 (10)	0.0252 (6)	0.178 (4)
H4B1	1.1998	0.5793	0.8655	0.03*	0.178 (4)
H4B2	1.2274	0.4649	0.7861	0.03*	0.178 (4)
C5B	1.0262 (2)	0.41072 (18)	0.86124 (16)	0.0222 (4)	0.178 (4)
H5B1	1.0822	0.3549	0.9018	0.027*	0.178 (4)
H5B2	0.9686	0.4545	0.9082	0.027*	0.178 (4)
C11	0.7060 (2)	0.13812 (17)	0.55819 (15)	0.0155 (4)	
C12	0.6196 (2)	0.20991 (19)	0.49657 (16)	0.0222 (4)	
H12	0.5488	0.2514	0.5273	0.027*	
C13	0.6355 (2)	0.22151 (19)	0.39194 (16)	0.0222 (4)	
H13	0.5776	0.2722	0.3521	0.027*	
C14	0.7355 (2)	0.15971 (18)	0.34479 (16)	0.0193 (4)	
H14	0.7461	0.1676	0.2727	0.023*	
C15	0.8197 (2)	0.08648 (19)	0.40339 (16)	0.0228 (4)	
H15	0.8877	0.0431	0.3713	0.027*	
C16	0.8051 (2)	0.07612 (18)	0.50916 (16)	0.0191 (4)	
H16	0.8639	0.0258	0.5487	0.023*	
C21	0.8152 (2)	0.03788 (17)	0.74897 (15)	0.0164 (4)	
C22	0.7770 (2)	−0.08818 (18)	0.70735 (16)	0.0199 (4)	
H22	0.688	−0.1188	0.6487	0.024*	
C23	0.8659 (2)	−0.16896 (18)	0.74967 (16)	0.0218 (4)	
H23	0.8371	−0.2543	0.7205	0.026*	
C24	0.9970 (2)	−0.12568 (19)	0.83469 (16)	0.0227 (4)	
H24	1.058	−0.1811	0.8642	0.027*	
C25	1.0383 (2)	−0.00127 (19)	0.87619 (17)	0.0239 (4)	
H25	1.1293	0.029	0.9334	0.029*	
C26	0.9474 (2)	0.08001 (18)	0.83467 (16)	0.0206 (4)	
H26	0.9757	0.165	0.865	0.025*	
C31	0.4685 (2)	0.35868 (17)	0.71326 (15)	0.0157 (4)	
C32	0.4624 (2)	0.45295 (19)	0.65030 (16)	0.0204 (4)	
H32	0.5452	0.5236	0.6677	0.024*	
C33	0.3364 (2)	0.4442 (2)	0.56256 (17)	0.0253 (5)	
H33	0.3344	0.5081	0.5197	0.03*	
C34	0.2143 (2)	0.3431 (2)	0.53752 (16)	0.0246 (5)	
H34	0.1288	0.337	0.4771	0.03*	
C35	0.2165 (2)	0.24971 (19)	0.60091 (16)	0.0227 (4)	
H35	0.1317	0.1805	0.5843	0.027*	
C36	0.3429 (2)	0.25774 (18)	0.68856 (16)	0.0191 (4)	
H36	0.3436	0.1941	0.7318	0.023*	
C41	0.5704 (2)	0.29021 (17)	0.91837 (15)	0.0163 (4)	
C42	0.4502 (2)	0.33272 (19)	0.96094 (16)	0.0207 (4)	
H42	0.4064	0.3973	0.9326	0.025*	
C43	0.3941 (2)	0.2821 (2)	1.04375 (17)	0.0257 (5)	
H43	0.312	0.3116	1.0713	0.031*	
C44	0.4581 (2)	0.1883 (2)	1.08629 (16)	0.0246 (4)	
H44	0.4195	0.1529	1.1427	0.03*	
C45	0.5785 (2)	0.14650 (19)	1.04613 (16)	0.0220 (4)	
H45	0.6231	0.0828	1.0756	0.026*	
C46	0.6347 (2)	0.19717 (18)	0.96279 (15)	0.0184 (4)	
H46	0.7175	0.168	0.9361	0.022*	
N1	0.75799 (16)	0.27932 (14)	0.76314 (12)	0.0155 (3)	
P1	0.67417 (5)	0.13242 (5)	0.69726 (4)	0.01549 (12)	
P2	0.64933 (5)	0.37383 (5)	0.81951 (4)	0.01544 (12)	

### Atomic displacement parameters (Å^2^)

**Table:**

	*U*^11^	*U*^22^	*U*^33^	*U*^12^	*U*^13^	*U*^23^
C1	0.0132 (8)	0.0182 (10)	0.0159 (10)	0.0018 (7)	0.0040 (7)	0.0010 (8)
C2	0.0180 (8)	0.0241 (11)	0.0195 (10)	0.0056 (8)	0.0058 (8)	0.0071 (8)
C3A	0.0296 (11)	0.0227 (12)	0.0311 (13)	−0.0010 (9)	0.0105 (9)	0.0010 (10)
C4A	0.0254 (12)	0.0214 (14)	0.0251 (14)	−0.0007 (10)	0.0044 (10)	−0.0016 (11)
C5A	0.0175 (9)	0.0259 (12)	0.0199 (11)	0.0022 (8)	0.0009 (8)	−0.0006 (9)
C3B	0.0296 (11)	0.0227 (12)	0.0311 (13)	−0.0010 (9)	0.0105 (9)	0.0010 (10)
C4B	0.0254 (12)	0.0214 (14)	0.0251 (14)	−0.0007 (10)	0.0044 (10)	−0.0016 (11)
C5B	0.0175 (9)	0.0259 (12)	0.0199 (11)	0.0022 (8)	0.0009 (8)	−0.0006 (9)
C11	0.0151 (8)	0.0143 (9)	0.0138 (9)	−0.0017 (7)	0.0014 (7)	−0.0009 (7)
C12	0.0241 (9)	0.0267 (12)	0.0176 (11)	0.0114 (8)	0.0041 (8)	0.0019 (9)
C13	0.0274 (10)	0.0220 (11)	0.0167 (10)	0.0080 (8)	0.0000 (8)	0.0045 (8)
C14	0.0207 (9)	0.0220 (11)	0.0127 (10)	−0.0017 (8)	0.0026 (7)	0.0034 (8)
C15	0.0233 (9)	0.0285 (12)	0.0206 (11)	0.0085 (9)	0.0095 (8)	0.0067 (9)
C16	0.0193 (9)	0.0230 (11)	0.0166 (10)	0.0066 (8)	0.0042 (8)	0.0058 (8)
C21	0.0194 (8)	0.0189 (10)	0.0141 (10)	0.0055 (8)	0.0078 (7)	0.0055 (8)
C22	0.0227 (9)	0.0192 (10)	0.0173 (10)	0.0018 (8)	0.0060 (8)	0.0022 (8)
C23	0.0304 (10)	0.0160 (10)	0.0224 (11)	0.0056 (8)	0.0124 (9)	0.0036 (8)
C24	0.0295 (10)	0.0242 (11)	0.0202 (11)	0.0129 (9)	0.0101 (9)	0.0082 (9)
C25	0.0279 (10)	0.0267 (12)	0.0170 (10)	0.0101 (9)	0.0011 (8)	0.0024 (9)
C26	0.0274 (10)	0.0179 (10)	0.0169 (10)	0.0068 (8)	0.0043 (8)	0.0017 (8)
C31	0.0157 (8)	0.0190 (10)	0.0141 (9)	0.0065 (7)	0.0051 (7)	0.0016 (8)
C32	0.0181 (9)	0.0226 (11)	0.0224 (11)	0.0051 (8)	0.0074 (8)	0.0049 (9)
C33	0.0265 (10)	0.0344 (13)	0.0210 (11)	0.0153 (9)	0.0079 (9)	0.0103 (9)
C34	0.0226 (9)	0.0375 (13)	0.0143 (10)	0.0151 (9)	0.0010 (8)	−0.0008 (9)
C35	0.0185 (9)	0.0248 (11)	0.0214 (11)	0.0041 (8)	0.0009 (8)	−0.0039 (9)
C36	0.0199 (9)	0.0191 (10)	0.0188 (10)	0.0049 (8)	0.0053 (8)	0.0017 (8)
C41	0.0150 (8)	0.0184 (10)	0.0131 (9)	0.0008 (7)	0.0018 (7)	−0.0005 (8)
C42	0.0193 (9)	0.0260 (11)	0.0183 (10)	0.0073 (8)	0.0053 (8)	0.0041 (9)
C43	0.0213 (9)	0.0368 (13)	0.0204 (11)	0.0066 (9)	0.0085 (8)	0.0026 (9)
C44	0.0259 (10)	0.0309 (12)	0.0150 (10)	−0.0019 (9)	0.0065 (8)	0.0032 (9)
C45	0.0274 (10)	0.0204 (11)	0.0155 (10)	0.0024 (8)	0.0008 (8)	0.0024 (8)
C46	0.0189 (9)	0.0198 (10)	0.0146 (10)	0.0027 (8)	0.0024 (7)	−0.0007 (8)
N1	0.0127 (7)	0.0162 (8)	0.0166 (8)	0.0007 (6)	0.0042 (6)	−0.0002 (7)
P1	0.0157 (2)	0.0163 (3)	0.0142 (3)	0.00285 (19)	0.00334 (18)	0.0017 (2)
P2	0.0146 (2)	0.0171 (3)	0.0149 (3)	0.00371 (19)	0.00380 (18)	0.0017 (2)

### Geometric parameters (Å, °)

**Table:**

C1—N1	1.499 (2)	C23—C24	1.388 (3)
C1—C2	1.547 (3)	C23—H23	0.95
C1—C5A	1.553 (3)	C24—C25	1.383 (3)
C1—H1	1	C24—H24	0.95
C2—C3A	1.530 (3)	C25—C26	1.393 (3)
C2—H2A	0.99	C25—H25	0.95
C2—H2B	0.99	C26—H26	0.95
C3A—C4A	1.546 (3)	C31—C36	1.396 (3)
C3A—H3A1	0.99	C31—C32	1.400 (3)
C3A—H3A2	0.99	C31—P2	1.842 (2)
C4A—C5A	1.512 (3)	C32—C33	1.390 (3)
C4A—H4A1	0.99	C32—H32	0.95
C4A—H4A2	0.99	C33—C34	1.378 (3)
C5A—H5A1	0.99	C33—H33	0.95
C5A—H5A2	0.99	C34—C35	1.394 (3)
C4B—H4B1	0.99	C34—H34	0.95
C4B—H4B2	0.99	C35—C36	1.393 (3)
C11—C16	1.392 (2)	C35—H35	0.95
C11—C12	1.400 (3)	C36—H36	0.95
C11—P1	1.847 (2)	C41—C46	1.391 (3)
C12—C13	1.381 (3)	C41—C42	1.403 (2)
C12—H12	0.95	C41—P2	1.8274 (19)
C13—C14	1.384 (3)	C42—C43	1.387 (3)
C13—H13	0.95	C42—H42	0.95
C14—C15	1.382 (3)	C43—C44	1.388 (3)
C14—H14	0.95	C43—H43	0.95
C15—C16	1.389 (3)	C44—C45	1.386 (3)
C15—H15	0.95	C44—H44	0.95
C16—H16	0.95	C45—C46	1.393 (3)
C21—C26	1.397 (3)	C45—H45	0.95
C21—C22	1.400 (3)	C46—H46	0.95
C21—P1	1.8388 (19)	N1—P1	1.7157 (17)
C22—C23	1.381 (3)	N1—P2	1.7205 (16)
C22—H22	0.95		
			
N1—C1—C2	114.93 (14)	C22—C23—H23	120
N1—C1—C5A	113.39 (15)	C24—C23—H23	120
C2—C1—C5A	105.15 (16)	C25—C24—C23	119.51 (18)
N1—C1—H1	107.7	C25—C24—H24	120.2
C2—C1—H1	107.7	C23—C24—H24	120.2
C5A—C1—H1	107.7	C24—C25—C26	120.43 (19)
C3A—C2—C1	104.78 (16)	C24—C25—H25	119.8
C3A—C2—H2A	110.8	C26—C25—H25	119.8
C1—C2—H2A	110.8	C25—C26—C21	120.72 (19)
C3A—C2—H2B	110.8	C25—C26—H26	119.6
C1—C2—H2B	110.8	C21—C26—H26	119.6
H2A—C2—H2B	108.9	C36—C31—C32	118.66 (17)
C2—C3A—C4A	103.07 (17)	C36—C31—P2	124.46 (14)
C2—C3A—H3A1	111.1	C32—C31—P2	116.80 (14)
C4A—C3A—H3A1	111.1	C33—C32—C31	120.67 (19)
C2—C3A—H3A2	111.1	C33—C32—H32	119.7
C4A—C3A—H3A2	111.1	C31—C32—H32	119.7
H3A1—C3A—H3A2	109.1	C34—C33—C32	120.24 (19)
C5A—C4A—C3A	103.38 (19)	C34—C33—H33	119.9
C5A—C4A—H4A1	111.1	C32—C33—H33	119.9
C3A—C4A—H4A1	111.1	C33—C34—C35	119.90 (18)
C5A—C4A—H4A2	111.1	C33—C34—H34	120.1
C3A—C4A—H4A2	111.1	C35—C34—H34	120
H4A1—C4A—H4A2	109.1	C36—C35—C34	120.09 (19)
C4A—C5A—C1	107.22 (17)	C36—C35—H35	120
C4A—C5A—H5A1	110.3	C34—C35—H35	120
C1—C5A—H5A1	110.3	C35—C36—C31	120.40 (18)
C4A—C5A—H5A2	110.3	C35—C36—H36	119.8
C1—C5A—H5A2	110.3	C31—C36—H36	119.8
H5A1—C5A—H5A2	108.5	C46—C41—C42	118.29 (17)
H4B1—C4B—H4B2	108.5	C46—C41—P2	123.86 (13)
C16—C11—C12	117.63 (17)	C42—C41—P2	117.40 (14)
C16—C11—P1	125.95 (14)	C43—C42—C41	121.13 (18)
C12—C11—P1	116.41 (13)	C43—C42—H42	119.4
C13—C12—C11	121.19 (17)	C41—C42—H42	119.4
C13—C12—H12	119.4	C42—C43—C44	119.87 (18)
C11—C12—H12	119.4	C42—C43—H43	120.1
C12—C13—C14	120.32 (18)	C44—C43—H43	120.1
C12—C13—H13	119.8	C45—C44—C43	119.67 (18)
C14—C13—H13	119.8	C45—C44—H44	120.2
C15—C14—C13	119.47 (18)	C43—C44—H44	120.2
C15—C14—H14	120.3	C44—C45—C46	120.47 (18)
C13—C14—H14	120.3	C44—C45—H45	119.8
C14—C15—C16	120.17 (17)	C46—C45—H45	119.8
C14—C15—H15	119.9	C41—C46—C45	120.55 (17)
C16—C15—H15	119.9	C41—C46—H46	119.7
C15—C16—C11	121.20 (17)	C45—C46—H46	119.7
C15—C16—H16	119.4	C1—N1—P1	121.43 (12)
C11—C16—H16	119.4	C1—N1—P2	115.22 (12)
C26—C21—C22	117.78 (17)	P1—N1—P2	121.76 (9)
C26—C21—P1	124.69 (15)	N1—P1—C21	104.70 (9)
C22—C21—P1	117.13 (14)	N1—P1—C11	102.52 (8)
C23—C22—C21	121.44 (19)	C21—P1—C11	101.65 (8)
C23—C22—H22	119.3	N1—P2—C41	104.71 (8)
C21—C22—H22	119.3	N1—P2—C31	104.59 (8)
C22—C23—C24	120.10 (19)	C41—P2—C31	100.62 (9)
			
N1—C1—C2—C3A	145.49 (16)	C42—C43—C44—C45	0.5 (3)
C5A—C1—C2—C3A	20.08 (18)	C43—C44—C45—C46	−0.6 (3)
C1—C2—C3A—C4A	−37.2 (2)	C42—C41—C46—C45	1.1 (3)
C2—C3A—C4A—C5A	40.1 (2)	P2—C41—C46—C45	173.20 (15)
C3A—C4A—C5A—C1	−27.7 (2)	C44—C45—C46—C41	−0.2 (3)
N1—C1—C5A—C4A	−121.37 (17)	C2—C1—N1—P1	103.43 (17)
C2—C1—C5A—C4A	4.99 (19)	C5A—C1—N1—P1	−135.56 (14)
C16—C11—C12—C13	−1.8 (3)	C2—C1—N1—P2	−62.41 (19)
P1—C11—C12—C13	179.38 (16)	C5A—C1—N1—P2	58.59 (18)
C11—C12—C13—C14	1.4 (3)	C1—N1—P1—C21	59.96 (15)
C12—C13—C14—C15	−0.2 (3)	P2—N1—P1—C21	−135.12 (10)
C13—C14—C15—C16	−0.7 (3)	C1—N1—P1—C11	−45.82 (15)
C14—C15—C16—C11	0.3 (3)	P2—N1—P1—C11	119.10 (10)
C12—C11—C16—C15	0.9 (3)	C26—C21—P1—N1	8.13 (17)
P1—C11—C16—C15	179.66 (15)	C22—C21—P1—N1	−179.28 (13)
C26—C21—C22—C23	0.5 (3)	C26—C21—P1—C11	114.56 (16)
P1—C21—C22—C23	−172.64 (14)	C22—C21—P1—C11	−72.86 (15)
C21—C22—C23—C24	−0.6 (3)	C16—C11—P1—N1	112.33 (17)
C22—C23—C24—C25	−0.3 (3)	C12—C11—P1—N1	−68.92 (16)
C23—C24—C25—C26	1.3 (3)	C16—C11—P1—C21	4.20 (19)
C24—C25—C26—C21	−1.4 (3)	C12—C11—P1—C21	−177.05 (15)
C22—C21—C26—C25	0.5 (3)	C1—N1—P2—C41	−135.34 (13)
P1—C21—C26—C25	173.05 (14)	P1—N1—P2—C41	58.87 (12)
C36—C31—C32—C33	−2.2 (3)	C1—N1—P2—C31	119.28 (13)
P2—C31—C32—C33	174.66 (14)	P1—N1—P2—C31	−46.52 (12)
C31—C32—C33—C34	1.0 (3)	C46—C41—P2—N1	20.87 (18)
C32—C33—C34—C35	0.5 (3)	C42—C41—P2—N1	−166.95 (15)
C33—C34—C35—C36	−0.9 (3)	C46—C41—P2—C31	129.18 (17)
C34—C35—C36—C31	−0.4 (3)	C42—C41—P2—C31	−58.64 (17)
C32—C31—C36—C35	1.9 (3)	C36—C31—P2—N1	76.55 (16)
P2—C31—C36—C35	−174.75 (14)	C32—C31—P2—N1	−100.16 (15)
C46—C41—C42—C43	−1.2 (3)	C36—C31—P2—C41	−31.86 (17)
P2—C41—C42—C43	−173.85 (16)	C32—C31—P2—C41	151.43 (14)
C41—C42—C43—C44	0.5 (3)		

### Hydrogen-bond geometry (Å, °)

**Table:**

Cg1 is the centroid of the C11–C16 ring.

**Table:**

*D*—H···*A*	*D*—H	H···*A*	*D*···*A*	*D*—H···*A*
C2—H2B···P2	0.99	2.82	3.269 (2)	108
C5A—H5A1···P2	0.99	2.82	3.202 (3)	104
C12—H12···Cg1	0.95	2.72	3.667 (2)	174
C3A—H3A2···Cg1^i^	0.99	2.89	3.811 (2)	155

Symmetry codes: (i) *x*+1, *y*, *z*.
